# Supplementary material for: Analysis of the evolution of resistance to multiple antibiotics enables prediction of the Escherichia coli phenotype-based fitness landscape
Source: PLoS Biol. 2022 Dec 13;20(12):e3001920. doi: 10.1371/journal.pbio.3001920 (PMC9746992; doi:10.1371/journal.pbio.3001920)

A: Parent in TET selection

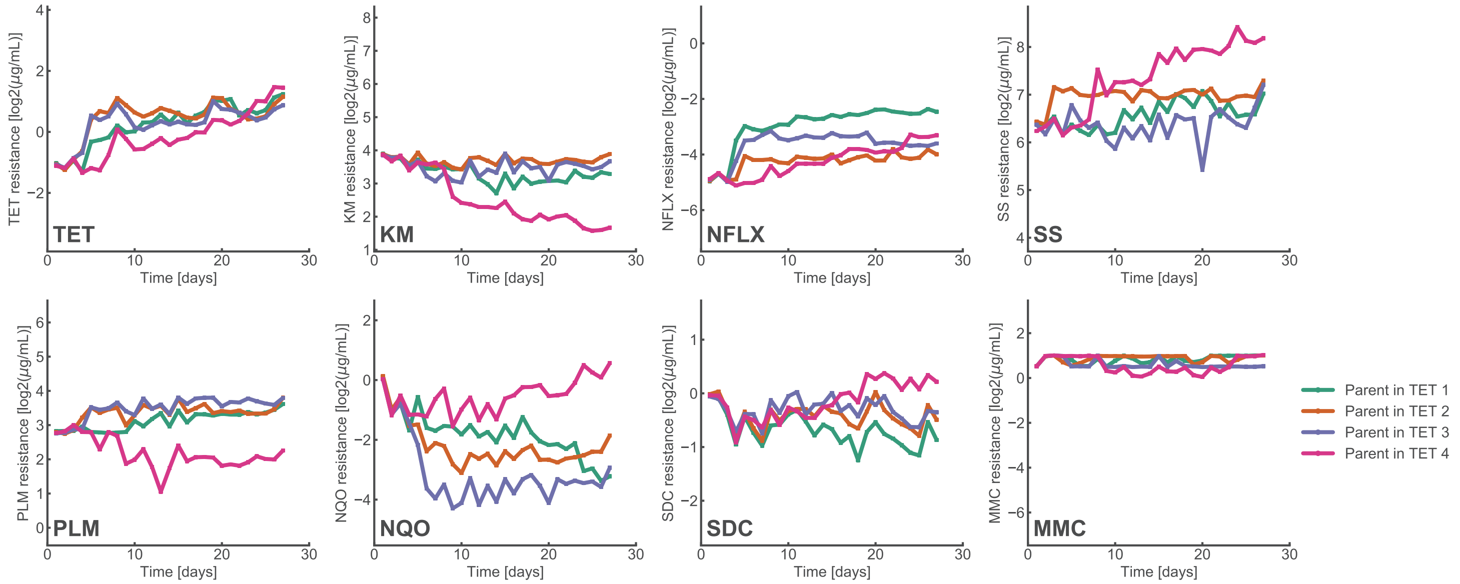

B: KME1 in TET selection

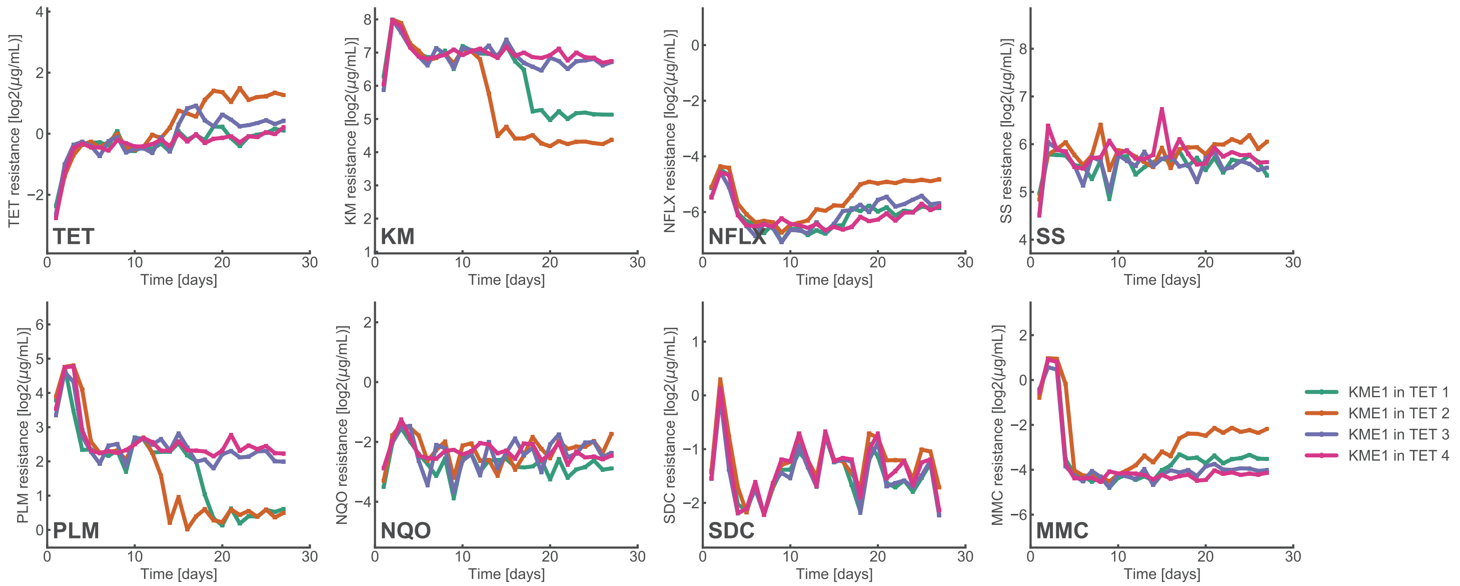

C: KME5 in TET selection

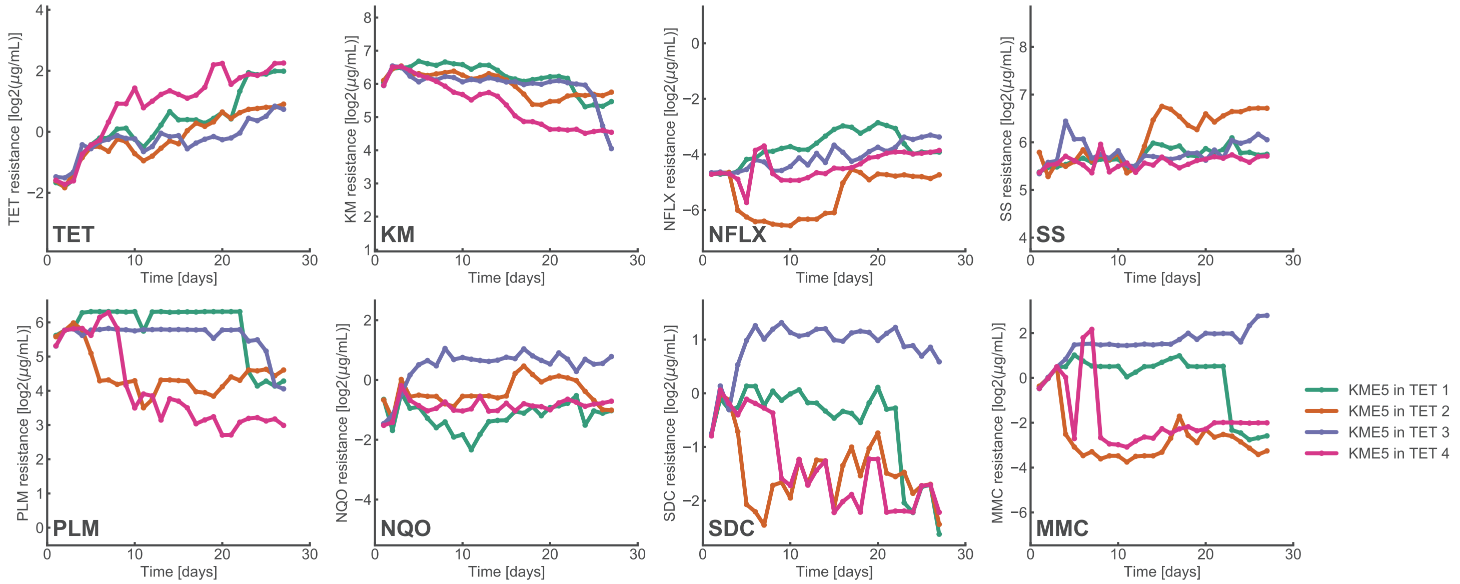

D: Parent in KM selection

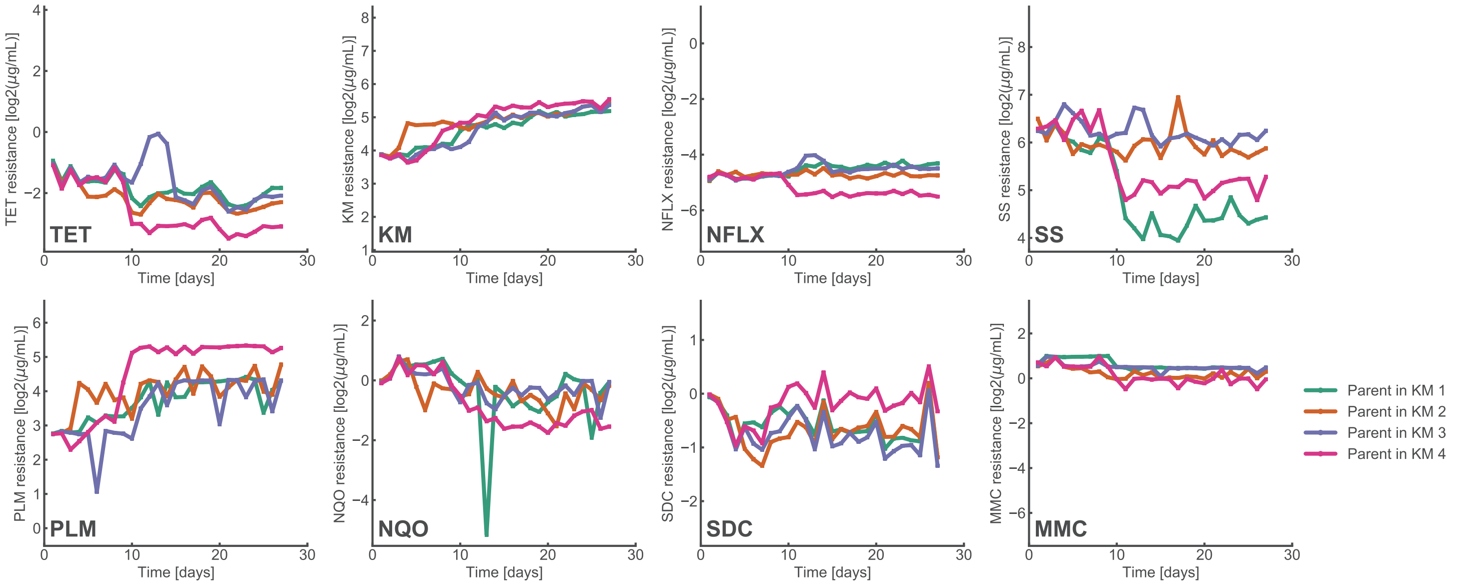

E: TETE4 in KM selection

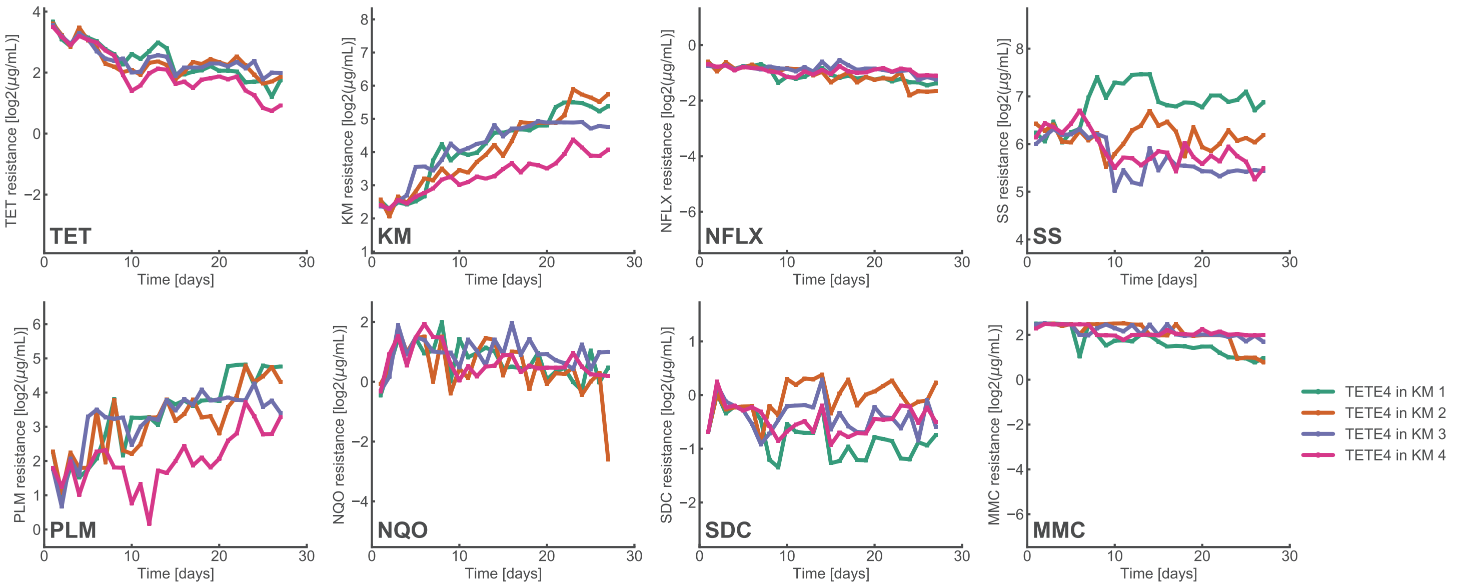

F: TETE6 in KM selection

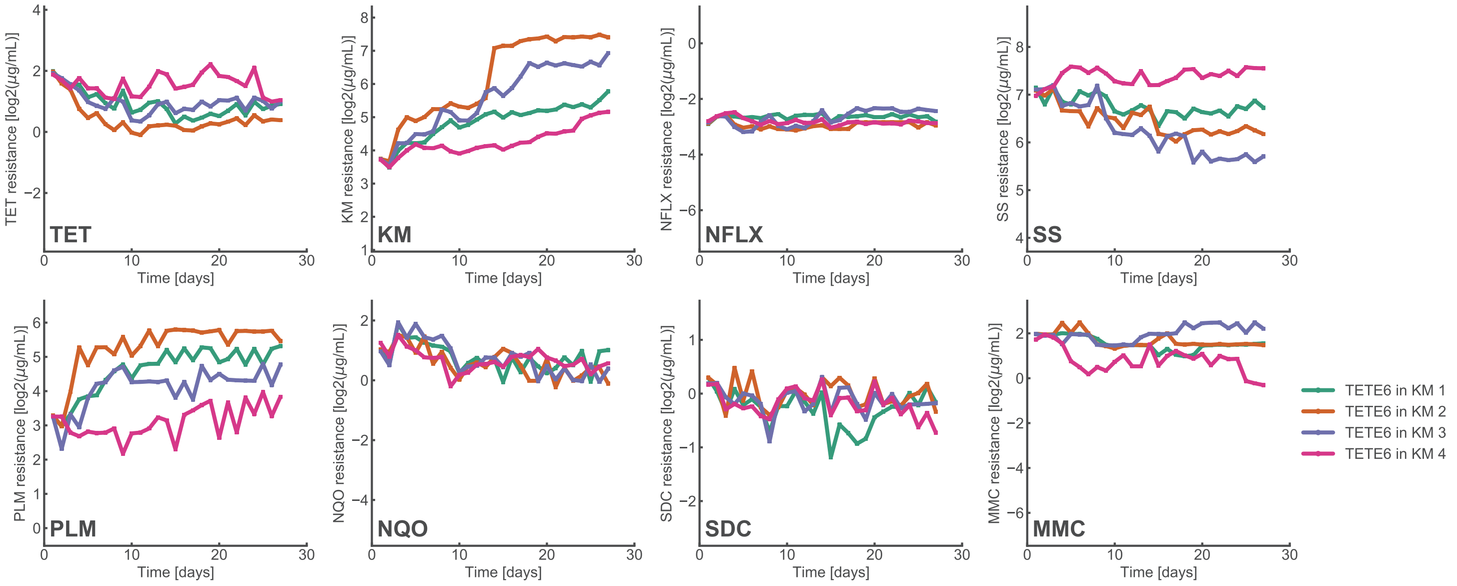

G: NFLX4 in KM selection

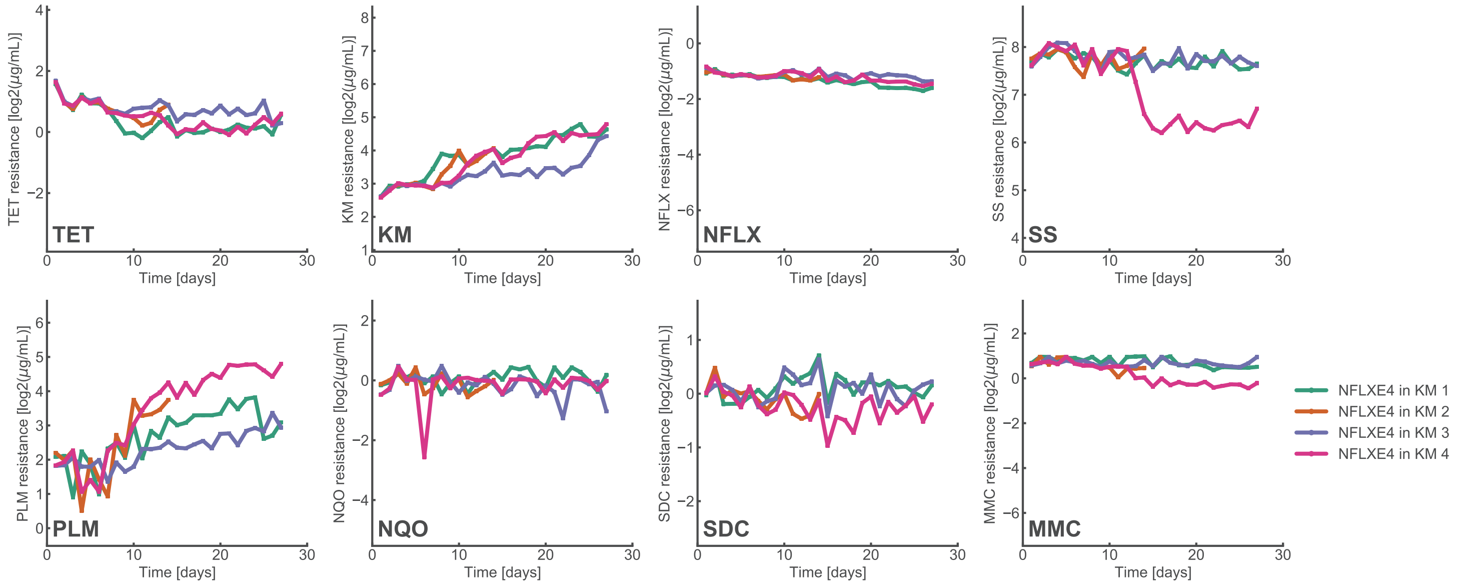

H: NFLX6 in KM selection

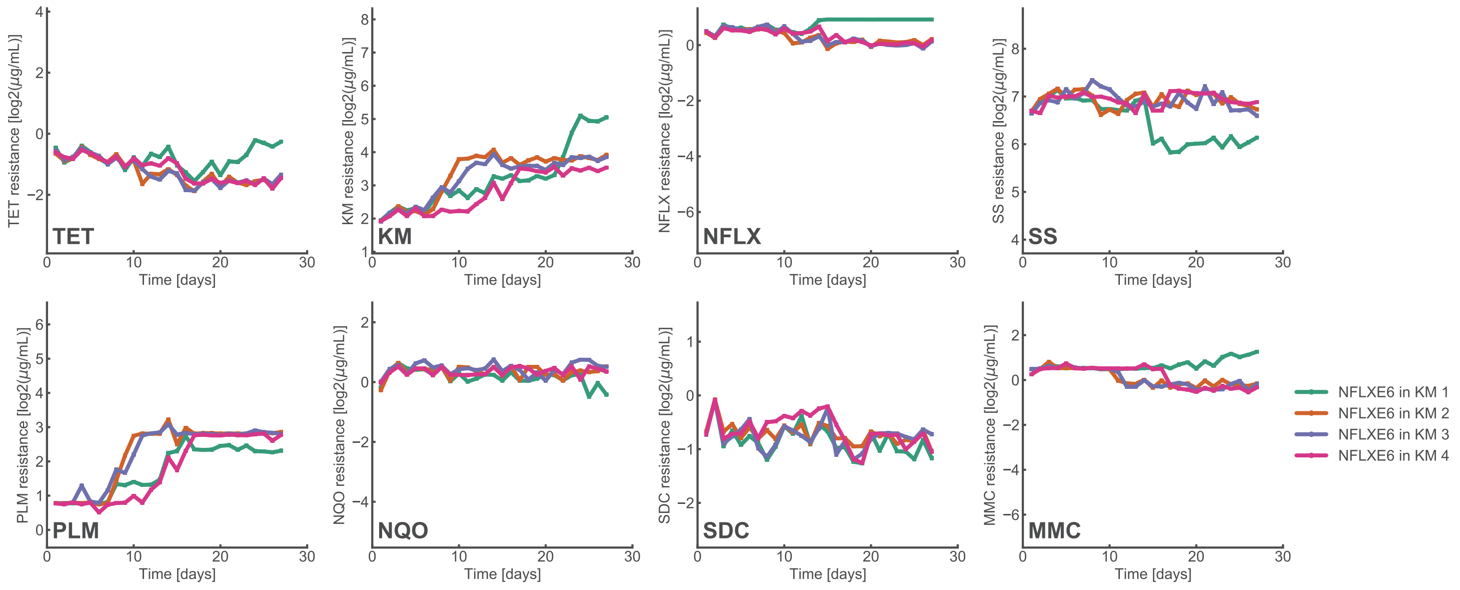

I: Parent in NFLX selection

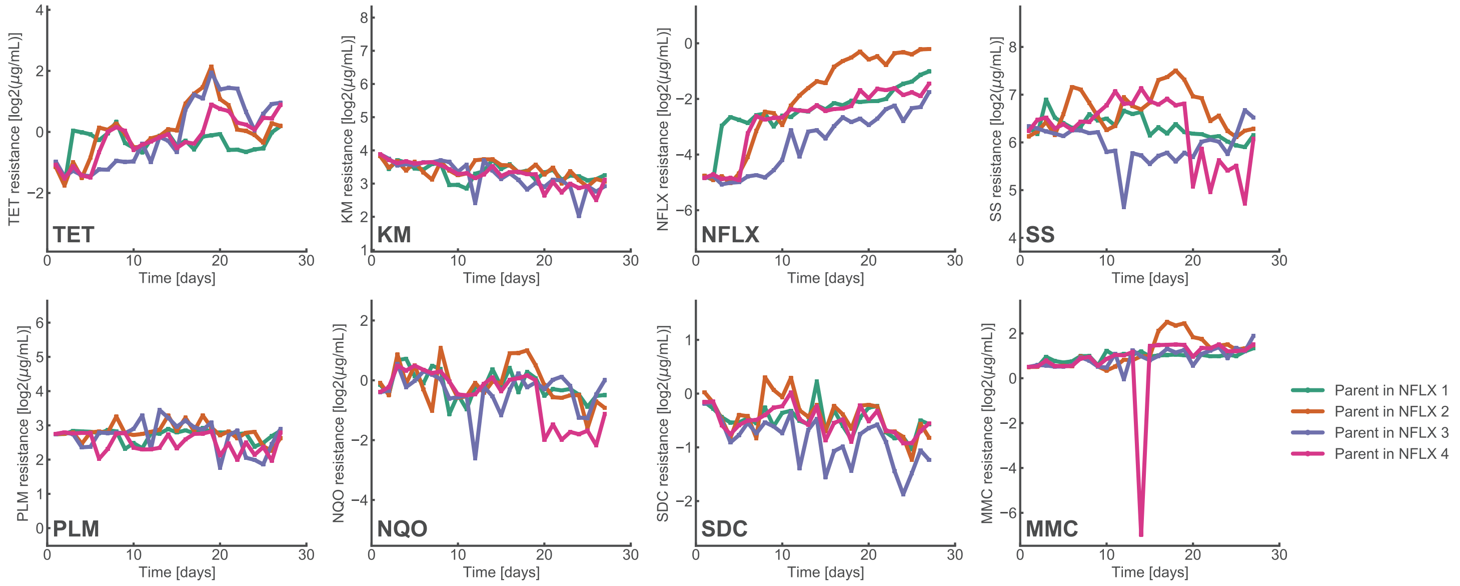

J: KME1 in NFLX selection

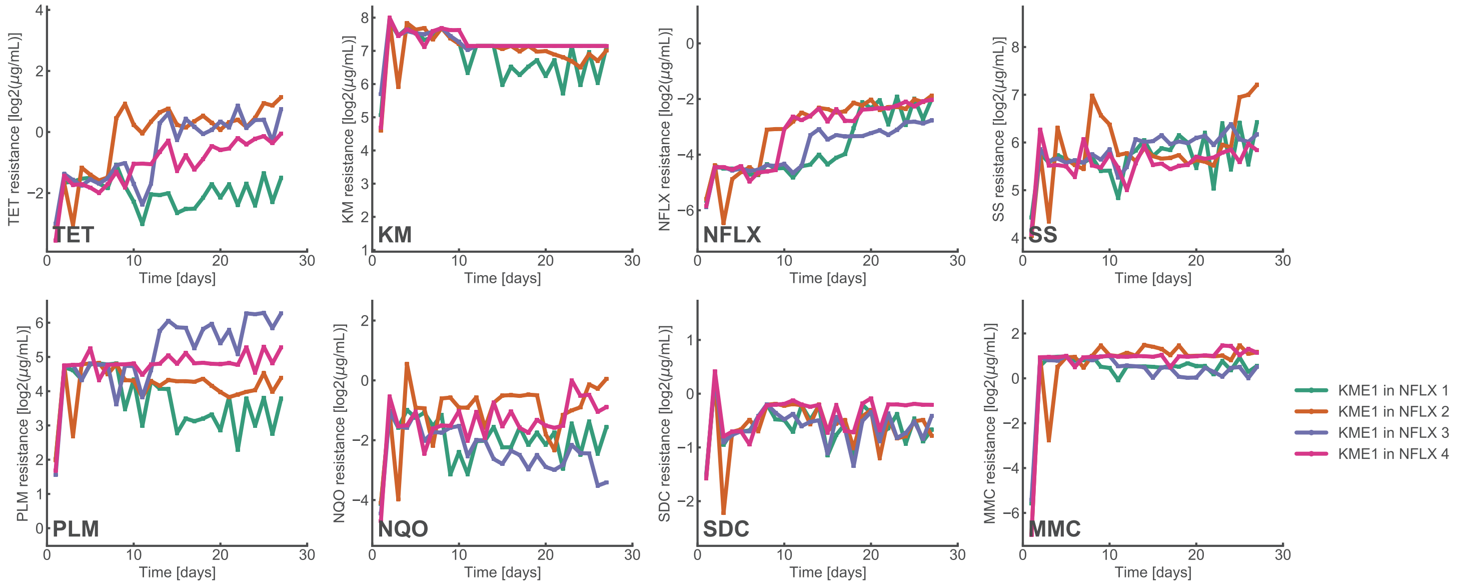

K: KME5 in NFLX selection

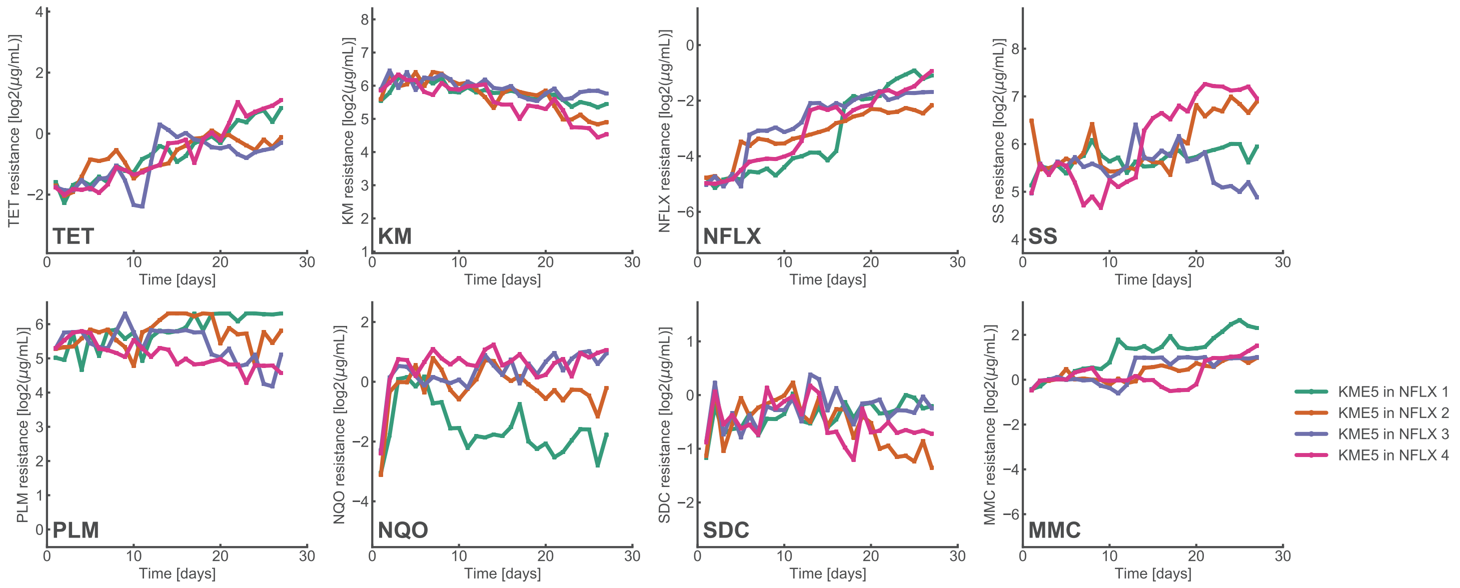

L: Parent in M9 (No selection)

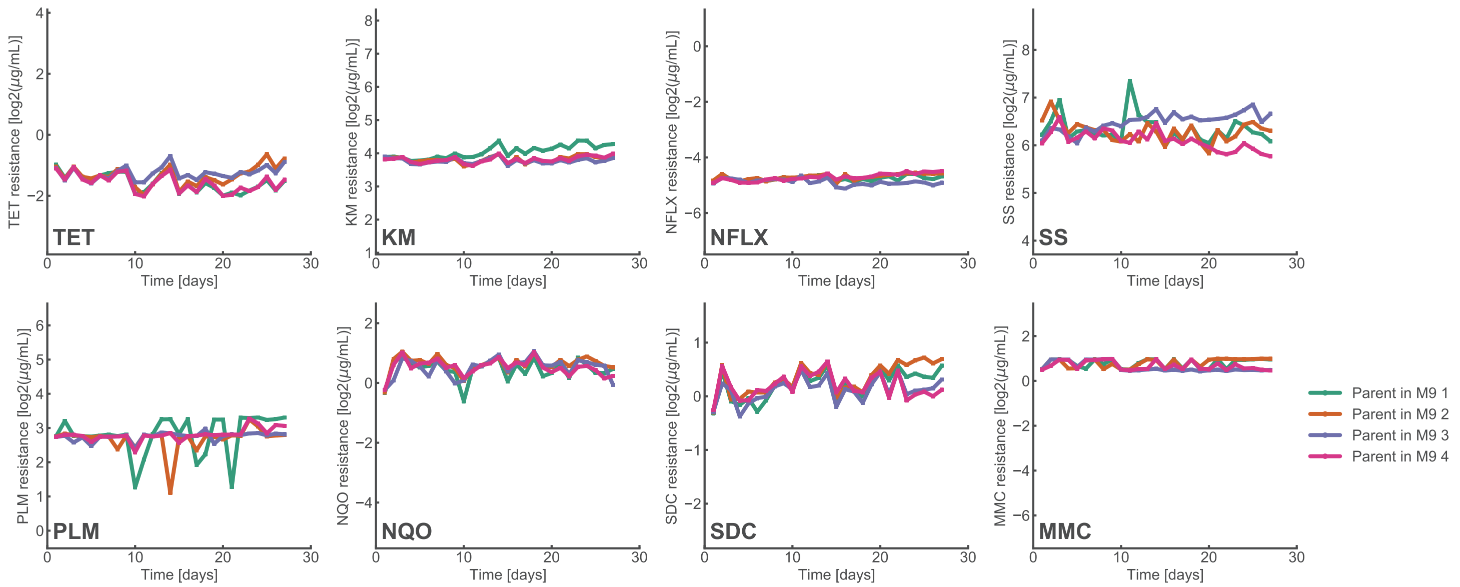

Supplement: S1 Fig — Data of four independent culture series are overlaid. The data underlying this figure can be found in S1 Data. (PDF) [file pbio.3001920.s001.pdf]
