# Supplementary figures and images for: Analysis of the evolution of resistance to multiple antibiotics enables prediction of the Escherichia coli phenotype-based fitness landscape
Source: PLoS Biol. 2022 Dec 13;20(12):e3001920. doi: 10.1371/journal.pbio.3001920 (PMC9746992; doi:10.1371/journal.pbio.3001920)

**A**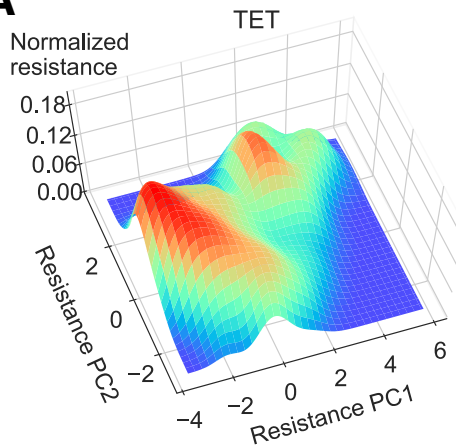**B**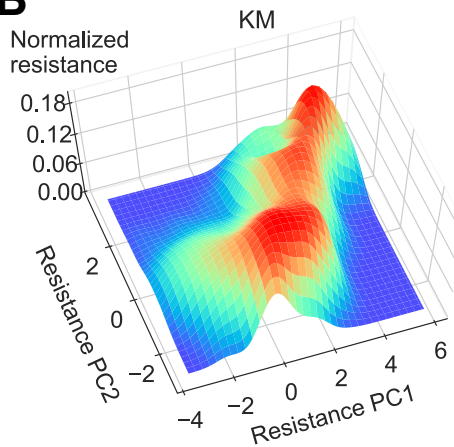**C**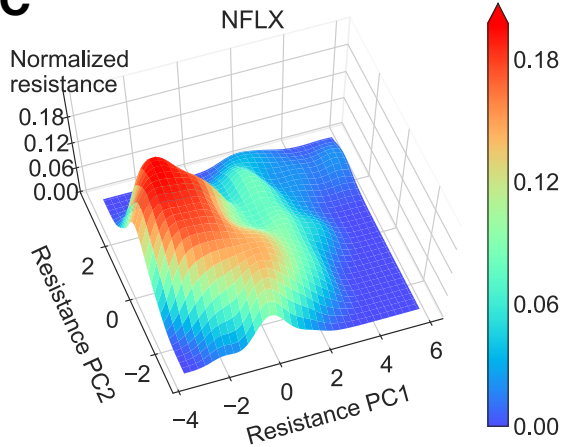**D**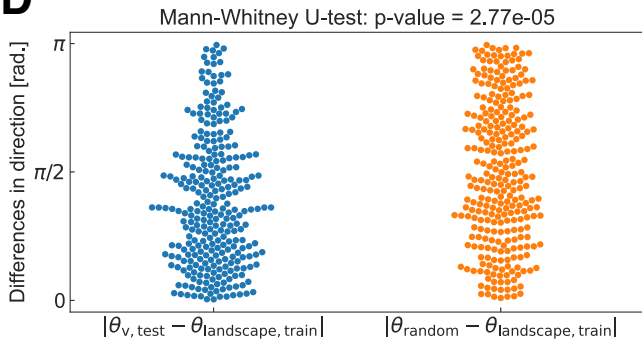

Supplement: S2 Fig — (A-C) The inferred phenotype-fitness landscapes using the training dataset. Here, the training dataset was obtained by randomly excluding one out of the four replicates for each experimental condition, resulting in a total of 33 lines. (D) Differences in the evolutionary directions between the simulated gradients from the phenotype-fitness landscape (θlandscape,train, inferred from the training dataset) and experimental observations (θv,test, based on the test dataset). Here, the test dataset contains the 11 hold-out samples that were not used in the training data. For comparison, the difference with a random direction (θrandom) sampled from a uniform distribution [0,2π) is also plotted. The data underlying this figure can be found in S1 Data. (PDF) [file pbio.3001920.s002.pdf]

**A**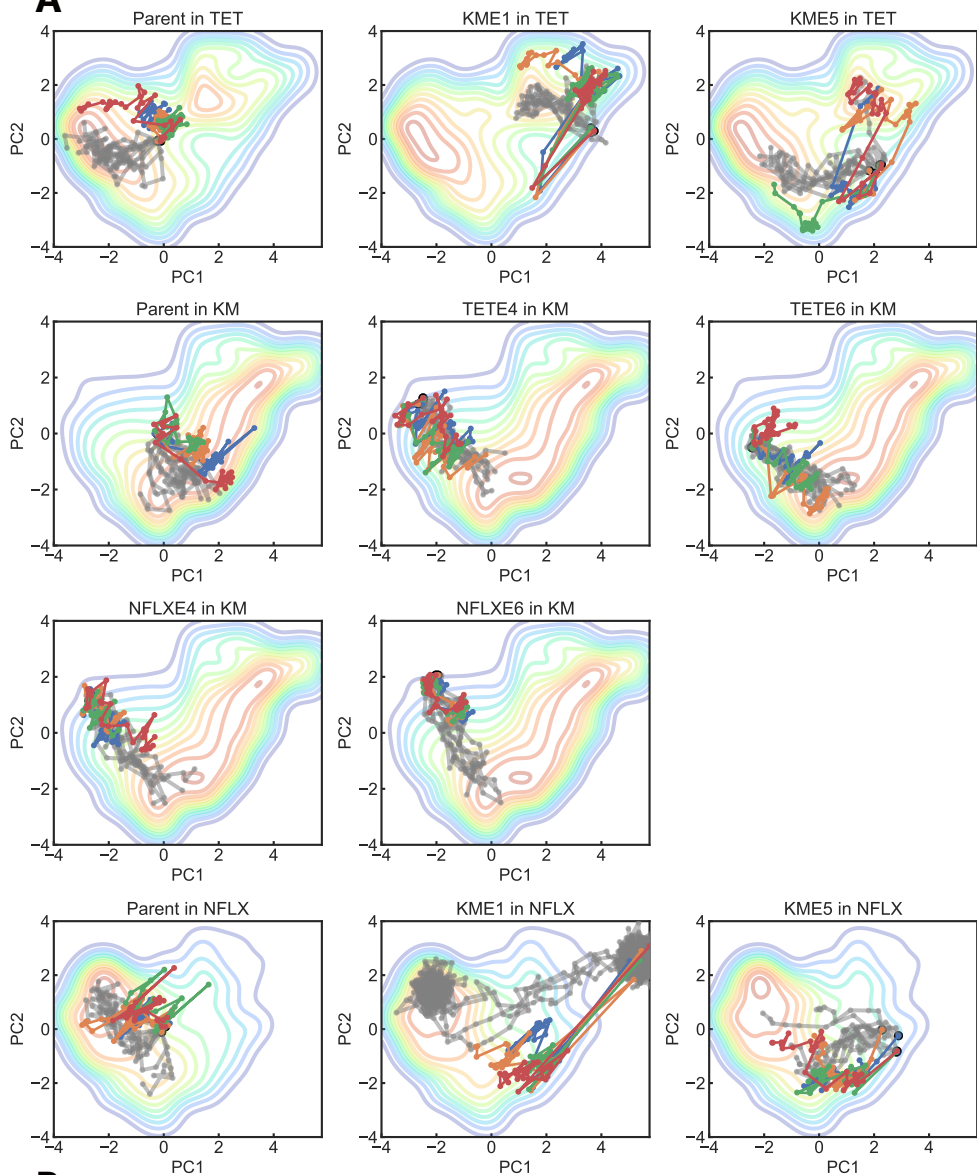**B**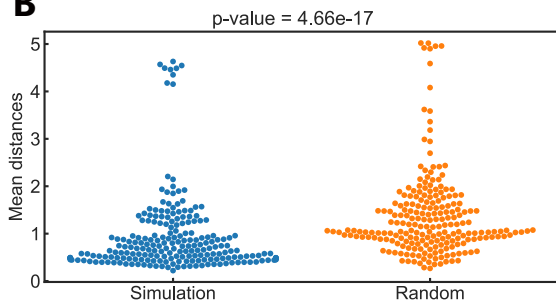

Supplement: S3 Fig — (A) The simulated trajectories of evolution (gray) overlayed on the experimental trajectories (blue, orange, green, and red) in the corresponding environments. The starting points were selected randomly from the four starting points of the experimental trajectories. The simulations were performed for 10 independent runs with 10 time steps, except for the simulations for KME1 in NFLX where we ran the simulation for 150 time steps in order to let the trajectories escape from a local optimum. (B) The mean distances L between the simulations and experimental trajectories (blue) and between random Brownian motion and experimental trajectories (orange). A total of 20 independent runs were performed for each environment, resulting into 20 × 11 = 220 estimates of L for the simulations and Brownian motion. The data underlying this figure can be found in S1 Data. (PDF) [file pbio.3001920.s003.pdf]

**A**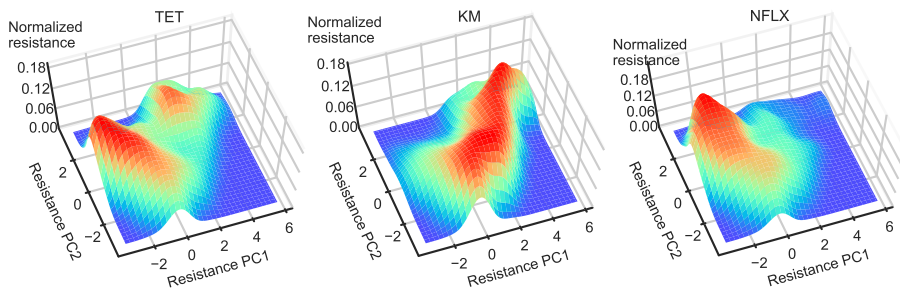**B**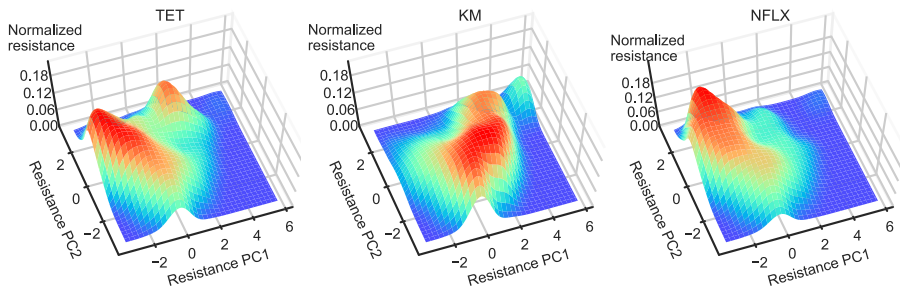**C**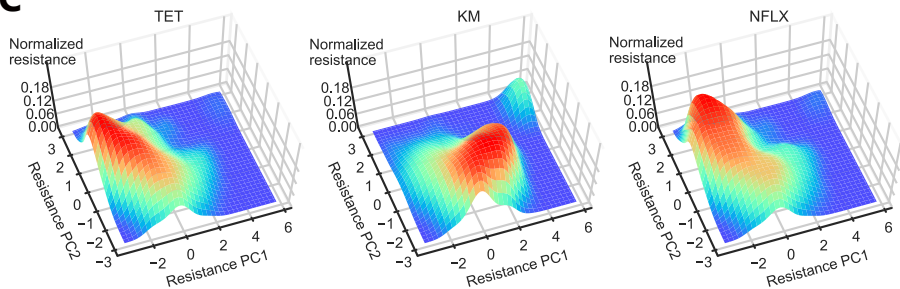

Supplement: S4 Fig — (A) Dataset of “Parent in TET,” (B) datasets of “Parent in TET” and “KME1 in TET” (8 trajectories), and (C) datasets of “Parent in TET,” “KME1 in TET,” and “KME5 in TET” (12 trajectories) were removed from the landmark inference, respectively. KME1 and KME5 strains evolved from independent culture series under the selection of the antibiotic, kanamycin (KM). The data underlying this figure can be found in S1 Data. (PDF) [file pbio.3001920.s004.pdf]
