# Supplementary material for: Analysis of the evolution of resistance to multiple antibiotics enables prediction of the Escherichia coli phenotype-based fitness landscape
Source: PLoS Biol. 2022 Dec 13;20(12):e3001920. doi: 10.1371/journal.pbio.3001920 (PMC9746992; doi:10.1371/journal.pbio.3001920)
Supplement: Attachment — Submitted filename: Response_to_Reviewers.docx [file pbio.3001920.s006.docx]

**Reply to Reviewer #1**

We would like to thank the reviewer for the constructive suggestions to improve our manuscript. We have revised the manuscript following these suggestions. Our responses to specific comments are shown below.

**Comment 0**: In the paper, Iwasawa et al. evolve multiple strains of E. coli in 8 different drugs, and construct PCA-based landscapes of drug resistance. These are then used to demonstrate the possibility of predicting and controlling evolution. The subject matter is topical and the approach seems promising. But there are some major issues to be addressed. Based on these, I cannot recommend publication in the present form.

**Response 0**: We thank the reviewer for his/her critical comments of our manuscript. Below, we present replies to the specific comments and revisions to improve the manuscript.

**Major points**

**Comment 1**: The term "fitness landscape" is usually reserved for genotype-fitness maps. Yet, this term has been used frequently to mean phenotype-fitness maps (or phenotype-fitness landscapes). This is potentially misleading and should be rectified throughout the paper.

**Response 1**: We thank the reviewer for the comment. Following this comment, we have replaced the phrase “fitness landscape” in the manuscript to “phenotype-fitness landscape” where it indicates the fitness landscapes based on phenotypes.

**Comment 2**: Am I right that the PCA was done by pooling together all the data from the 44 experiments? If so, how robust are the properties of the phenotype-fitness landscapes to the inclusion of new datasets? For example, if one were to remove the lines evolved in TET, would one have qualitatively different landscapes and different predicted dynamics for the other drugs? This is important since the correspondence between theta_v and theta_landscape does not appear too strong in Fig 4I.

**Response 2**: Yes. The PCA was done using all data from the 44 trajectories. Here we would like to answer the question on the robustness of landscape estimation using the example of the TET landscape. The TET landscape has a double peak structure (Fig 4A), which is supported mainly by the eight trajectories of the “KME1 in TET” and “KME5 in TET” dataset. In S4 Fig, we show how the landscape structure changes when we remove the above datasets under TET selection from the landscape inference. While the double peak structure was conserved in S4 Fig A and S4 Fig B in which a part of dataset was removed, the minor peak disappeared in S4 Fig C where data in all trajectories under TET selection were removed. These results suggest that the properties of the phenotype-fitness landscape could change with the inclusion of new datasets, such as trajectories starting from evolved strains under other drugs. Because this susceptibility is caused by the lack of sufficient data, a more robust landscape is expected to be obtained through the collection of a sufficient number of experimental conditions and replicates. It should also be noted that the structure of the TET landscape is rather conserved when we only remove the four replicates of “Parent in TET” strains, suggesting that the landscape structure in our study is robust to small changes to the dataset.

To clarify the content given above, we have added S4 Fig to the revised manuscript and the following sentences to the Discussion:

**Revision 2: Discussion, p12**

A related limitation of this study is that the properties of the inferred landscapes could be susceptible to the inclusion of new data, such as evolution time-series under other drugs. For example, the TET landscape has a double peak structure (Fig 4A), which is supported mainly by the eight trajectories of the “KME1 in TET” and “KME5 in TET” datasets. In S4 Fig, we show how the landscape structure changes when we remove the dataset under TET selection from the landscape inference. While the double peak structure is conserved in S4 Fig A and S4 Fig B, in which a part of datasets was removed, the minor peak in S4 Fig C disappeared when data in all trajectories under TET selection were removed. These results suggest that the properties of the phenotype-fitness landscape can change with the inclusion of new datasets, such as trajectories starting from evolved strains under other drugs. Because this susceptibility is caused by the lack of sufficient data, a more robust landscape is expected to be obtained through the collection of a sufficient number of experimental conditions and replicates, which should be addressed in future studies.

**S4 Fig.** The inferred phenotype-based fitness landscapes when the datasets under tetracycline (TET) selection are removed. (A) dataset of “Parent in TET”, (B) datasets of “Parent in TET” and “KME1 in TET” (8 trajectories), and (C) datasets of “Parent in TET,” “KME1 in TET,” and “KME5 in TET” (12 trajectories) were removed from the landmark inference, respectively. KME1 and KME5 strains evolved from independent culture series under the selection of the antibiotic, kanamycin (KM).

**Comment 3**: In a similar vein, the fact that the PCA was done for the entire set of time series and then used to predict properties of the same time-series appears somewhat circular. I understand that the point here is that a dimensionally-reduced representation still retains considerable information about the system. But the predictive power of the approach would be convincing if the training and testing datasets were distinct. For example, if two out of the four replicates were chosen randomly from each combination to obtain the landscapes, how good would the predictions be for the remainder of the datasets?

**Response 3**: We thank the reviewer for the comment on the predictive power of the approach in this study, which is an important issue. Although train/test splitting for validating predictability is an effective method, we have not used splitting in our study due to the small data size. We compared the landscape directions with experimental values without splitting in Fig 4I, and this was used to validate the landscape inference method.

Here, following the reviewer’s suggestion, we validated the predictive power of the approach by splitting the whole dataset to the training/test dataset. We obtained the training dataset by randomly excluding one out of the four replicates for each experimental condition, resulting in a total of 33 lines; the remaining 11 hold-out samples were used as the test dataset. We inferred the phenotype-fitness landscape using the training data (S2 Fig A–C), and compared the evolutionary directions (θ_landscape, train_) with experimental observations based on the test data (θ_v, test_) (S2 Fig D). The number of samples decreased by a factor of 1/4 compared to Fig 4I, but we could still observe a significant correspondence between the landscape-based predictions and the experimental observations from the test data compared to the control of the random data. Note that the comparison with random is not a powerful validation as in the reviewer’s fourth comment, and further experimental validations with different conditions are an important direction for further work. Overall, this analysis suggests the predictive power of the inferred landscapes for evolutionary directions.

We added a paragraph to the “Inference of the phenotype-fitness landscapes” subsection to discuss the findings and validation of the predictability of the landscapes.

**Revision 3: Results (Inference of the phenotype-fitness landscapes), p7**

The predictability of the inferred landscapes was evaluated by applying train/test splitting to the whole dataset (S2 Fig). Here, the training dataset was obtained by randomly excluding one out of the four replicates for each experimental condition, resulting in a total of 33 lines, and the excluded 11 lines were used for the test dataset. The phenotype-fitness landscape was inferred using the training data (S2 Fig A–C), and was compared with the evolutionary directions (θ_landscape, train_), with experimental observations based on the test data (θ_v, test_) (S2 Fig D). The landscape gradients and experimental observations showed significant agreement. Note that this verification for predictability is only possible for the landscape of TET, KM, and NFLX since the laboratory evolution experiments were performed only under these three drugs. However, the landscape properties of the other five drugs might also have similar predictive power due to the tight interconnectedness of drug resistance acquisition relations, which is known as cross-resistance and collateral sensitivity [11,12,14,18]. Overall, the results suggest a correspondence between the landscape-based properties and the experimental observations, indicating the validity and predictive power of the inferred landscapes.

**S2 Fig.** The inferred phenotype-fitness landscape and its validation based on the train/test splitting of the dataset. (A)–(C) The inferred phenotype-fitness landscapes using the training dataset. Here, the training dataset was obtained by randomly excluding one out of the four replicates for each experimental condition, resulting in a total of 33 lines. (D) Differences in the evolutionary directions between the simulated gradients from the phenotype-fitness landscape (θ_landscape, train_, inferred from the training dataset) and experimental observations (θ_v, test_, based on the test dataset). Here, the test dataset contains the 11 hold-out samples that were not used in the training data. For comparison, the difference with a random direction (θ_random_) sampled from a uniform distribution [0, 2π) is also plotted.

**Comment 4**: There are a few issues with the section "Utilizing landscapes for controlling evolution". Firstly, the "agreement" between the simulated and experimental trajectories in Fig S2A is not convincing (the fact that the match is better than random is not very useful). Given the high noise and sample-to-sample variation in the experimental data, I am not sure why this exercise is necessary in the first place. The results in Fig 5 can stand on their own. Secondly, the authors say that "Of course, real trajectories might deviate from the simulation given here because mutations accumulate during the course of evolution, and these mutations can change the structure of the landscape... However, a deviation is something we should be pleased with since it could provide novel insights into evolutionary constraints and their relations with the strain's genetic (or non-genetic) background." Here the authors seem to be suggesting that their method can be used as a tool for generating novel insights from unexpected system behavior, which is at odds with the goal of controlling evolution based on existing knowledge. Overall, the message of this section is not clear.

**Response 4.1:** We thank the reviewer for the critical comments. In response to the first point, S2 Fig A in the original manuscript (S3 Fig A in the revised manuscript) was displayed to validate the simulation parameters, which were estimated directly from experimental values to verify the following simulations in the manuscript. Although the comparison with random is not a powerful method, the analysis supported the validity of the results obtained from our simulations, considering that the simulated and experimental trajectories showed qualitative agreement.

To clarify this point, we have modified the manuscript as follows:

**Revision 4.1: Results. (Utilizing landscapes for controlling evolution), p10**

In S3 Fig A, we show the simulated trajectories compared to the corresponding experimental trajectories to validate the estimated simulation parameters. With some exceptions (e.g., KME5 under TET selection), the simulation results show a qualitative agreement with the experimental trajectories.

**Response 4.2:** For the second point, we apologize for the clarity of the text in the manuscript. Although we believe the landscapes could be a powerful prediction tool, the landscape structure could be vulnerable to new datasets as discussed in Comment 2. The construction of a more robust landscape can be obtained through the extensive investigation of experimental conditions and replicates, which could be addressed in future work. We expect that such robust landscapes will be applicable for predicting evolution.

To clarify this message, we have modified the manuscript as follows:

**Revision 4.2:** **Results. (Utilizing landscapes for controlling evolution), p10**

Note that the inferred phenotype-fitness landscapes were constructed based on laboratory experiments with a limited number of genetic backgrounds and thus, evolutionary trajectories starting from different genetic backgrounds might deviate from these landscapes since the different combination of mutations could alter the landscape structure [47,48]. Such alterations in the landscape structure could be detected through discrepancies between the original inferred landscape and experimental trajectories, leading to novel insights of evolutionary constraints, their relations with the strain's genetic (or non-genetic) background, and the construction of a more robust phenotype-fitness landscape.

**Minor points**

**Comment 5**: "Phenotypes" used in the paper are the principal components that resolve the variation in IC_50 in an 8-dimensional space. Of course, the exact contribution to the principal components of biologically interpretable phenotypes is unknown. For clarity, the sense in which "phenotype" is used in this paper (i.e. principal components) should be stated early on in the paper, perhaps even in the abstract.

**Response 5**: We thank the reviewer for the suggestion. Following the reviewer’s suggestion, we have modified the introduction as follows so that it is clarified that we have used principal components as phenotypes.

**Revision 5: Introduction, p2**

The critical feature of our work is that we used eight different antibiotic resistance values (IC_50_) as probes for fitness, and their principal components as phenotypes (Fig 1B).

**Comment 6**: In the abstract, the authors say "we inferred that the fitness landscape underlies the evolution dynamics of resistance". Perhaps they mean "we inferred the fitness landscape that underlies the evolution dynamics of resistance"?

**Response 6**: We thank the reviewer for the suggestion. We have modified the abstract as a result of the other reviewer’s comments and have removed the corresponding sentence from the manuscript.

**Comment 7**: In Results, "eight drugs other than antibiotics" should be "five drugs other than antibiotics".

**Response 7**: We thank the reviewer for the comment. Following this suggestion, we have modified the main text as follows:

**Revision 7: Results (Laboratory evolution with multi-antibiotic resistance measurements), p2**

In addition to TET, KM, and NFLX, we adopted the following five drugs (other than antibiotics): sodium salicylate …

**Comment 8**: In Figs 3A and 3B, the end-states of the strains should also be marked in circles (of a different color). Same applies for other figures showing trajectories. It would also help to mark the maxima in Fig 4A in Figs 3A and 3B.

**Response 8**: We thank the reviewer for the suggestion. We have modified Figs 2A, 2B, and Figs 3A, 3B so that the end-states of the states are clarified. We have also added black triangles to Figs 3A, 3B to show the maxima in Figs 4A, 4B.

**Fig. 3. Evolutionary trajectories in a two-dimensional principal component analysis (PCA) space.**

A total of 44 trajectories (11 conditions with four replicates each) in an eight-dimensional resistance space were projected into two-dimensional space using PCA. The variance explained by the first two components was 41% and 23%, respectively. (A) Evolutionary trajectories under tetracycline (TET) selection. Trajectories starting from the parent and kanamycin (KM)-evolved strains are highlighted by colors, while the gray lines show the trajectories in other conditions. The black and yellow circles represent the state of starting strains and the strains on the last day, respectively. (B) Evolutionary trajectories under KM selection. Trajectories starting from the parent and TET-evolved strains under KM selection are highlighted in color. The black triangles in (A) and (B) show the peaks of the inferred phenotype-fitness landscape for TET and KM, respectively, and will be explained later in Fig 4.

**Reply to Reviewer #2**

We would like to thank the reviewer for the constructive suggestions to improve our manuscript. We have revised the manuscript following these suggestions. Our responses to specific comments are shown below.

**Comment 0**: The manuscript by Iwasawa et al. addresses an important problem in the field of antibiotic resistance evolution. Being able to predict evolutionary trajectories leading to resistance is one of the common goals in the field but has been proven to be extremely difficult due to several intrinsic difficulties such as stochastic events, size of combinatorial space, unknown genotypes, and epistasis. Authors came up with an interesting approach where they utilize the entire evolutionary trajectories of 7 evolving cultures (with 4 replicates for each) under antibiotic selection. In their analysis, they primarily use phenotypic changes to construct adaptive fitness landscapes. The paper is generally very well written and clear. Experiments and analyses are well designed and performed. I have the following major criticisms.

**Response 0**: We thank the reviewer for his/her positive evaluation of our manuscript. Below, we present replies to the specific comments and revisions to improve the manuscript.

**Comment 1**: Authors carry out a PCA to construct landscapes shown in Figure 4. These are interesting landscapes but they look artificially smooth and uniform. In our experience, landscapes are almost always rugged dampening our prediction capabilities. I wonder whether the smooth landscapes are resulting due to the moving average used as described in page 6: "As the raw evolutionary trajectories were subject to experimental noise, we applied a moving average of six time points to the trajectories using a triangular window." Will the landscapes look different or less predictable if no averaging was done? Also, why the window size of 6 was chosen?

**Response 1**: We thank the reviewer for the comment on the smoothness of the landscape. The phenotype-fitness landscape was calculated based on the raw trajectories, not the smoothed trajectories. Thus, the smoothness is not related to the window size, but stems from the “grid size” for discretizing the resistance values and the “bandwidth” of the Gaussian kernel. The grid size was defined through a heuristic method called the Freedman–Diaconis rule and the bandwidth was defined so that the mean squared error between the inferred landscape and the normalized resistance values were minimized, as described in the Materials and Methods.

To show that the bandwidth is an important factor controlling the ruggedness of the landscape, we show the inferred landscape using h = 0.3 as the bandwidth in Fig R1 (shown below; h = 0.47 is used for the landscapes in the main text). The shorter the bandwidth, the more rugged the landscape becomes, but such landscapes were not adopted due to the discrepancies with experimental resistance values. Because the number of mutations is small compared with the frequency of the experimental noise in the resistance time series (Fig 1C), it is fair to say that our experimental system cannot detect the ruggedness of the genotype-phenotype map.

To clarify this point, we have added the following sentences to the manuscript:

**Revision 1: Results (Inference of the phenotype-fitness landscapes), p6**

The inferred landscapes have a small number of peaks for each landscape, suggesting low ruggedness [4,7]. Note that the high noise frequency in the original IC_50_ time series, compared with the mutation acquisition frequency, implies that the noise stems from measurement errors rather than from genotypic ruggedness.

As to the reviewer’s question of the window size, the window size of 6 days was selected so that the experimental noise (frequency of 2–3 days^-1^) for resistance measurements could be sufficiently suppressed, while the overall trend could be conserved.

**Fig R1.** The phenotype-fitness landscape inferred from the experimental data using h = 0.3 for the bandwidth of the Gaussian kernel

**Comment 2**: The predicted landscapes shown in Figs 4 and 5 have the axis of PC1 and PC2. I understand why it is done but I did not understand what PC1 and PC2 physically/biologically mean? Do these PCs correspond to anything meaningful/helpful?

**Response 2**: We thank the reviewer for the question regarding the interpretation of the principal components. Because the principal components are constructed using the resistance (IC_50_) values, one naive way to interpret them would be through the resistance values. From Fig 3, we can speculate that PC1 is correlated/anticorrelated with TET, NQO, SS, NFLX, and KM. Similarly, from Fig 3J, it can be observed that PC2 is related to PLM and MMC resistance.

As for the gene expressions underlying the principal components, we can analyze the relationship between the principal component scores of the starting strains (Parent, KME1, KME5, TETE4, TETE6, NFLXE4, NFLXE6) and their gene expression levels which were measured in our previous study (Maeda *et al*., 2020). By extracting the genes that had high correlations with the principal components, we found that *acrA* (R = -0.52) and *hemB* (R = -0.57) expression seem to be correlated with the PC1 score, where *acrA* was related to TET and NQO resistance through the AcrAB-TolC efflux pump. Mutations in *hem* have been found for aminoglycoside resistance in previous studies (Suzuki *et al*., 2014). *lon* (R = 0.76) and *ogt* (R = -0.55) expression seem to be correlated with PC2, where both genes have been reported to be related to MMC resistance (Maeda *et al*., 2020). The fact that *lon* expression is correlated with PC2 score is consistent with the results of the landscape analysis, where we report that *lon* mutations are responsible for the minor peak in the TET landscape. These correlation analyses support our initial interpretations of the principal components. However, since this was a primitive correlation analysis based on a small number of data points and statistical evaluation was difficult, more concrete analysis for the biological inferences of the principal components should be performed in future studies.

To clarify that the principal components could be interpreted through the drug resistance values, we have modified the manuscript as follows:

**Revision 2: Results (Observing trajectories in the PCA space), p5**

For example, the phenotypes with high KM resistance were located on the lower-right side of the PCA space, indicating that PC1 is positively correlated with KM resistance (Fig 3D). This tendency is also consistent with the evolutionary trajectories under KM selection (Fig 3B).

**Comment 3**: Evolution of resistance can be predicted and even manipulated in some very special cases. But in most cases, even the very first evolutionary step (which is almost random) such a resistance conferring mutation can steer evolution towards very unpredictable directions. I like the analysis and findings of this manuscript but there is need for further description of the limitations of authors' approach. Particularly, there are several assumptions made for creating fitness landscapes. These should be articulated in the discussion.

**Response 3**: We thank the reviewer for the comment. As the reviewer points out, there are resistance-conferring mutations with a wide spectrum of variations. If these various mutations lead to different directions of phenotypic changes (i.e., high dimensionality of the phenotype-fitness map), it will be difficult to predict them precisely using the phenotype-fitness landscape. However, previous laboratory evolution studies suggest high repeatability and a rather deterministic structure, implying the low dimensionality of the phenotype-fitness map. For such cases, our proposed method could be effective.

To clarify this point, we modified the discussion as follows:

**Revision 3: Discussion, p12**

Indeed, mutations conferring drug resistance have a wide spectrum and if these various mutations lead to different directions of phenotypic changes (i.e., high dimensionality of the phenotype-fitness map), it would be difficult to predict them precisely using the phenotype-fitness landscape in this study. However, previous laboratory evolution studies suggest high repeatability and a rather deterministic structure, implying the low dimensionality of the phenotype-fitness map [11,14-16,18]. For such cases, the phenotype-fitness landscape should be capable of predicting drug resistance evolution.

**Reply to Reviewer #3**

We would like to thank the reviewer for the constructive suggestions to improve our manuscript. We have revised the manuscript following these suggestions. Our responses to specific comments are shown below.

**Comment 0**: This is a review of "Multi-dimensional antibiotic resistance time series reveals the phenotype-based fitness landscape for Escherichia coli." This paper presents a large set of well-designed experiments and clever analyses to understand the fitness tradeoffs that come along with drug resistant mutations. These tradeoffs are then exploited to predict how the fitness of mutants will change when exposed to different drugs. The ideas in this paper are novel and the conclusions are well supported by the data. Overall, I think this is a super cool study and it's probably the best paper I've read all year.

My major comments are mainly to address the clarity of the study. I hope they help the authors make this nice paper even better.

**Response 0**: We thank the reviewer for his/her positive evaluation of our manuscript. Below, we present replies to the specific comments and revisions to improve the manuscript.

**Comment 1**: The title is very jargony. The phrase 'multi-dimensional' is particularly vague in both the title and the abstract. I eventually realized that this refers to the multiple drugs and multiple time points. Maybe something more like: "Ultra-rich data of how fitness changes across environments and over time reveals fitness landscapes that predict evolution."

**Response 1**: We thank the reviewer for the comment on the phrase “multi-dimensional”. We have modified the abstract as follows to clarify the notion of “multi-dimensional”.

**Revision 1: Abstract, p1**

Herein, we address this problem by inferring the phenotype-based fitness landscape for antibiotic resistance evolution by quantifying the multi-dimensional phenotypic changes, i.e., time-series data of resistance for eight different drugs.

**Comment 2**: Also, I recommended combining the following two sentences in the abstract into a single clearer sentence: "Herein, we address this problem by inferring the fitness landscape for antibiotic resistance evolution by quantifying the phenotypic changes, that is, multi-dimensional time-series measurements of the drug resistance profile. Using the time-series data of drug resistance for multiple drugs, we inferred that the fitness landscape underlies the evolution dynamics of resistance." I recommend the following instead: "Herein, we address this problem by inferring the fitness landscape for antibiotic resistance evolution by quantifying time-series data of drug resistance for multiple drugs"

**Response 2**: We thank the reviewer for the suggestion. Following the suggestion, we have modified the corresponding sentences in the abstract as follows, and deleted the second sentence.

**Revision 2: Abstract, p1**

Herein, we address this problem by inferring the phenotype-based fitness landscape for antibiotic resistance evolution by quantifying the multi-dimensional phenotypic changes, i.e., time-series data of resistance for eight different drugs.

**Comment 3**: The introduction is usually clearly and beautifully written. However, I did have trouble with this sentence: "Parallel laboratory evolution experiments have shown that most single nucleotide and amino acid changes have varying patterns and low repeatability, implying the existence of multiple paths in genotypic space to reach a fitted phenotype." How about something like this instead, "Parallel laboratory evolution experiments have shown that different single nucleotide changes can underlie similar phenotypic changes, implying the existence of multiple paths in genotypic space to reach a fitted phenotype."

**Response 3**: We thank the reviewer for the suggestion. We have modified the corresponding sentence as the reviewer has suggested.

**Revision 3: Introduction, p2**

Parallel laboratory evolution experiments have shown that different single nucleotide changes can underlie similar phenotypic changes, implying the existence of multiple paths in the genotypic space to reach a fitted phenotype [14,31-33].

**Comment 4**: Figure 1A and 1B were very confusing for me. I recommend the following changes.

a. I thought 1A was describing an evolution experiment because it is labeled "laboratory evolution with…". But now I realize panel 1B actually describes the evolution. Can you switch the order so that 1B is first (make 1B the new 1A). This would help because first you do the evolution, and then at every(?) timepoint of the evolution you do the cross-resistance test.

b. Can you rename 1A (which I think should actually be 1B) with a title that makes clear this is NOT the setup of the evolution experiment, but rather the set-up of the cross-resistance measurements?

c. Can you provide more details of the set up of the evolution experiment in the text? You mention 7 starting strains, maybe also mention how many generations there are between every timepoint, and how you transfer the cells from one timepoint to the next, and whether this was done in flasks, tubes, or plates? I know this is all in the methods, but it helps to have a little bit more in the main text. It would certainly have helped me in that I would not have confused the evolution experiment with the cross-resistance test.

d. By the time I got to 1C I understood and wrote on the figure, "wow, this is cool!"

**Response 4.a,b**: We thank the reviewer for the kind suggestion. We have switched the order of Figs 1A, 1B as suggested. We have also revised the title for Fig 1B (which was originally Fig 1A) as follows, so that the setup of the cross-resistance measurements becomes clearer:

**Revision 4.b: Caption of Figure 1b, p3**

Schematic figure describing the protocol of the laboratory evolution experiment with simultaneous IC_50_ measurements along the evolutionary trajectories in the present study.

**Response 4.c**: Following the reviewer’s comment 4c, we have added the details of the laboratory evolution experiment to the Results section as follows:

**Revision 4.c: Results (Laboratory evolution with multi-antibiotic resistance measurements), p2**

The daily passage occurred in 384-well microplates for every ~10 generations using an automated culture system (see Materials and Methods).

Fig 1. **Laboratory evolution of *Escherichia coli* to probe evolutionary trajectories in a multi-dimensional resistance space.**

(A) The experimental conditions used in the current study. We prepared seven starting strains, i.e., the parent *E. coli* strain, evolved under TET (TETE4, TETE6), evolved under KM (KME1, KME5), and evolved under NFLX (NFLXE4, NFLXE6). Starting from these strains, we performed laboratory evolution under three drugs (TET, KM, and NFLX). (B) Schematic figure describing the protocol of the laboratory evolution experiment with simultaneous IC_50_ measurements along the evolutionary trajectories in the present study. For every passage, IC_50_ was quantified for eight drugs, and cells were transferred from the well with the highest selection-drug concentration where the cell concentration exceeded a threshold. (C) The time series of IC_50_ values during the evolution of the KME5 strain under TET, for example. Data of four independent culture lines are overlaid. The resistance values for tetracycline (TET), kanamycin (KM), norfloxacin (NFLX), sodium salicylate (SS), phleomycin (PLM), 4-nitroquinoline 1-oxide (NQO), sodium dichromate dihydrate (SDC), and mitomycin C (MMC) are shown. All time-series data in the present study are presented in S1 Fig.

**Comment 5**: I like figure 2 and agree with the conclusion that mechanisms are modular for KM and NFLX.

**Response 5**: We thank the reviewer for the compliment.

**Comment 6**: In figure 3 I made a note about the KM evolved strains finding a different peak than the parent strain. I was happy to see that this is addressed later in the manuscript.

**Response 6**: We thank the reviewer for the comment. To make this point clearer, we have added the following sentences to the “Observing trajectories in the PCA space” subsection.

**Revision 6: Results (Observing trajectories in the PCA space), p4**

While most of the strains took similar trajectories, indicating convergence in the phenotypic space, several conditions (such as strains under TET evolution starting from KME1 and KME5) exhibited different evolutionary paths (Fig 3A). This divergence for TET evolution and the underlying genetic backgrounds will be discussed later.

**Comment 7**: In figure 4, two of the axes are the same axes as those in figure 3. But they have slightly different names. In figure 3 they are labeled "PC1" and in 3 they are labeled "resistance PC1". Can you use the same names to make clearer that these are the same things?

**Response 7**: We thank the reviewer for the suggestion. Following this suggestion, we have revised the axes in Fig 3 so that the axes with the same meaning all have the same name, i.e., “Resistance PC1, 2”.

**Comment 8**: I was confused about which conditions were analyzed in the manner depicted by figure 4I. Since you did evolutions in KM and TET only, does this analysis only work on those conditions? In other words, the landscapes are only being climbed for TET and KM. In the other cases, since there was no evolution performed, you should not expect any climbing to happen, just moving around. If I've interpreted this correctly, perhaps make clearer in the results section that this only applies to some conditions.

**Response 8**: As the reviewer points out, the verification of the landscapes was performed only for the TET, KM, and NFLX landscapes since the laboratory evolution experiments were performed only under these three drugs. However, the landscape properties of the other five drugs might also have similar predictive power due to the tight interconnectedness of drug resistance acquisition relations, which is known as cross-resistance and collateral sensitivity. The validity of the other landscapes should be addressed in future studies.

We have added the following sentences to the manuscript to clarify this point:

**Revision 8: Results (Inference of the phenotype-fitness landscapes), p7**

Note that this verification for predictability is only possible for the landscape of TET, KM, and NFLX since the laboratory evolution experiments were performed only under these three drugs. However, the landscape properties of the other five drugs might also have similar predictive power due to the tight interconnectedness of drug resistance acquisition relations, which is known as cross-resistance and collateral sensitivity [11,12,14,18].

**Comment 9**: Figure 5 and the simulations are very clever. Predicting and driving evolution like this is a big deal. I hope you plan to perform similar experiments in the wet lab. That would be quite the trick! (though totally unnecessary for this manuscript, which is already a major contribution to the field).

**Response 9**: We thank the reviewer for the comment. We do believe that utilizing the landscapes to perform simulations and predictions for experimental evolution will be the next large step. We are planning such experiments, and we hope we will be able to see the consequences soon.
